# Supplementary material for: Enterprise digital transformation’s impact on stock liquidity: A corporate governance perspective
Source: PLoS One. 2024 Mar 20;19(3):e0293818. doi: 10.1371/journal.pone.0293818 (PMC10954117; doi:10.1371/journal.pone.0293818)
Supplement: S1 Appendix — (ZIP) [file pone.0293818.s001.zip › S1 Appendix/S1 Appendix/Description of indicators and data.docx]

**Sample data description**

1.The sample period of our research is the annual data of Chinese listed companies from 2012 to 2021. It is worth noting that some of the data used in this study includes the actual data of each company from 2011 to 2022.

2. We excluded financial and insurance companies from the scope of analysis. During our research, we deleted sample data from ST or *ST companies and any invalid sample data during the research period. In addition, to avoid extreme values in the data, we conducted a 1% and 99% trimming process on the data in the study.

3. To avoid outliers and explain the differences between enterprises, the government size is converted using natural logarithms. Considering the time lag and extreme values of raw data in the impact of enterprise digital transformation on stock liquidity, the duration is advanced by one year and amplified by 100 times when calculating stock liquidity. This processing ensures the reliability and robustness of theoretical and empirical evidence.

**Data sources**

The original data of enterprise digital transformation, namely the annual report of listed companies, comes from Cninfo (website: <http://www.cninfo.com.cn/)>.

Raw data of stock liquidity (the number of days of annual trading days of enterprise stocks, the amount of stock transactions on enterprise trading days, and the rate of return of reinvestment considering cash dividends on enterprise trading days), Fund guarantee, Enterprise growth, Ownership concentration, Business investment opportunity, Zeros index, Roll index, Bid-ask Spread,Afq, and Spcr,from CSMAR database (website: [https://data.csmar.com/)](https://data.csmar.com/)。).

Enterprise scale, Asset liquidity, Profitability, Financial leverage, and Stock return, from Wind Database Terminal(website: <https://www.wind.com.cn/)>.

Financing constraint, Internal control, Information disclosure, Financial technology, from MARK Database(website: <https://www.macrodatas.cn/>).

**Variable construction:**

| Variables | Variable name | Variable Symbol | Variable label |
| --- | --- | --- | --- |
| **Y** | Stockliquidity(Liquidity) | Liquidity | Take the opposite number in accordance with the Amihud method; the greater the value, the higher the stock's liquidity. Method:  $\mathrm{ILLIQ}_{i,t}=\frac{1}{D_{i,t}}\sum_{d=1}^{D_{i,t}} \sqrt{\frac{\left\vert r_{i,t,d} \right\vert}{V_{i,t,d}}}{;\mathrm{Liquidity}}_{i,t}=-\mathrm{ILLIQ}_{i,t+1}$ |
| **X** | Enterprise digitization(DIG) | DIG | Word frequency statistics of enterprise digital feature words based on text analysis.Reference the word bank of enterprise digital transformation keywords constructed by Zhao et al(2021). |
| **Mechanism** | Financing constraint | FC | Refers to the SA index constructed by Hadlock and Pierce.SA=0.043×Size2-0.737×Size-0.04×Age |
|  | Internal control | IC | The Dibo internal control index. |
|  | Information disclosure | ID | Refer to research results Bharath and Pasquariello, using principal component analysis to calculate. |
| **Controls** | Enterprise scale | Size | Take the natural log of total assets |
|  | Asset liquidity | Cur | Current assets ratio: end-of-term current assets / ending current liabilities |
|  | Profitability | Roe | Roe : Net profit / shareholders' equity balance |
|  | Financial leverage | Lev | Asset-liability ratio: Total liabilities / Total assets |
|  | Fund guarantee | Netcash | Net cash flow per share: The ratio of the inflow of cash and cash equivalents of the Company minus the outflow balance (net inflow or net expenditure) to the total share capital of the Company |
|  | Enterprise growth | Growth | Revenue growth rate: (Amount of operating income current year-amount of previous year of operating income) / amount of operating income previous year |
|  | Earnings per share | Eps | Earnings per share: The ratio of after-tax profit to the total number of shares |
|  | Ownership concentration | Top10 | Share of top 10 shareholders: The sum of the shareholding ratio of the top ten shareholders |
|  | Business investment opportunity | Tobin Q | Tobin Q: Total market value / assets |
| **Robustness** | Zeros index | Zeros | Zeros index: Days of zero yield / days of trading |
|  | Roll index | Roll | Roll index =$\left\{ \begin{aligned} \sqrt[2]{-cov(\Delta p_{t},\Delta p_{t-1})} \\ 0 \end{aligned} \right.$ / Average daily transaction amount of the year. $\Delta p_{t}$Consider the daily yield of the cash dividend reinvestment for a single stock in the t cycle.$cov (\Delta p_{t},\Delta p_{t-1})$In the t cycle, the first-order difference of the daily yield of cash dividend reinvestment is considered. |
|  | Bid-ask Spread | Bas | The ratio of the difference between the sell price and the purchase price and the midpoint of the sell and the purchase price. |
|  | DIG1 | DIG1 | Word frequency statistics of enterprise digital feature words based on text analysis.Reference the word bank of enterprise digital transformation keywords constructed by Wu et al(2021). |
|  | DIG2 | DIG2 | Percentage of the total number of keywords in the digital transformation of enterprises in the total number of text words in the annual report of listed companies. |
| **Endogeneity** | DIG.IV | DIG.IV | The average value of enterprise digitalization across industries and regions serves as the instrumental variable. |
|  | PSM | PSM | This study divided the control group and the experimental group based on the digital transformation of enterprises is greater than the average value. |
| **Heterogeneity** | Financial technology | Financial technology | The total search volume of financial technology-related keywords in the same region is obtained by summing the number of search results and taking the natural logarithm. |
|  | Financial market | Financial market | Adopt Fan Gang's marketization index report |
|  | Digital policy guidance | Digital policy guidance | The Action Outline for Promoting the Development of Big Data issued by the Chinese government in 2015. |
| **Further analysis** | Afq | Afq | $\mathrm{Afq}_{i,j,t}=(-1)*\frac{\left\vert\mathrm{FORECAST}_{i,j,t}-\mathrm{EPS}_{i,j,t} \right\vert}{\mathrm{PRICE}_{i,j,t-1}}$  FORECAST is the analyst's prediction for the company's earnings per share. EPS is the actual earnings per share of the company. PRICE is the company's standardized stock price. |
|  | Spcr | Spcr | Referring to Jung et al. (2023), the NCSKEW index was used to measure the stock price crash risk to test the economic effect. |
