# Supplementary material for: Enterprise digital transformation’s impact on stock liquidity: A corporate governance perspective
Source: PLoS One. 2024 Mar 20;19(3):e0293818. doi: 10.1371/journal.pone.0293818 (PMC10954117; doi:10.1371/journal.pone.0293818)
Supplement: S1 Appendix — (ZIP) [file pone.0293818.s001.zip › S1 Appendix/S1 Appendix/The digital transformation feature word bank.docx]

**1.The digital transformation feature word bank(Zhao et al., 2021)**

Data management, data mining, data network, data platform, data center, data science, digital control, digital technology, digital communication, digital network, digital intelligence, digital terminal, digital marketing, digital, big data, cloud computing, cloud IT, cloud ecology, cloud services, cloud platform, blockchain, Internet of Things, machine learning, mobile Internet, industrial Internet, industrial Internet, Internet solutions Internet technology, Internet thinking, Internet action, Internet business, Internet mobile, Internet application, Internet marketing, Internet strategy, Internet platform, Internet model, Internet business model, Internet ecology, e-commerce, e-commerce, Internet, "Internet+", online and offline, online to offline, online and offline, O2O, B2B, C2C, B2C, C2B, artificial intelligence, high-end intelligence Industrial intelligence, mobile intelligence, intelligent control, intelligent terminal, intelligent mobility, intelligent management, intelligent factory, intelligent logistics, intelligent manufacturing, intelligent storage, intelligent technology, intelligent equipment, intelligent production, intelligent networking, intelligent system, intelligence, automatic control, automatic monitoring, automatic detection, automatic production, numerical control, integration, integration, integrated solution, integrated control, integrated system Industrial cloud, future factory, intelligent fault diagnosis, life cycle management, manufacturing execution system, virtualization, virtual manufacturing, information sharing, information management, information integration, information software, information system, information network, information terminal, information center, informatization, networking, industrial information, industrial communication

**2.The digital transformation feature word bank（Wu et al., 2021）**

Artificial intelligence, business intelligence, image understanding, investment decision support system, intelligent data analysis, intelligent robots, machine learning, deep learning, semantic search, biometrics, face recognition, voice recognition, identity verification, automatic driving, natural language processing, big data, data mining, text mining, data visualization, heterogeneous data, credit reporting, augmented reality, hybrid reality, virtual reality, cloud computing Stream computing, graph computing, memory computing, multi-party security computing, brain like computing, green computing, cognitive computing, fusion architecture, billion level concurrency, EB level storage, Internet of Things, information physical system, blockchain, digital currency, distributed computing, differential privacy technology, smart financial contracts, mobile Internet, industrial Internet, mobile Internet, Internet health, e-commerce, mobile payment, third-party payment, NFC payment Smart energy, B2B, B2C, C2B, C2C, O2O, Internet connection, smart wear, smart agriculture, smart transportation, smart medical, smart customer service, smart home, smart investment advisor, smart culture and tourism, smart environmental protection, smart grid, smart marketing, digital marketing, unmanned retail, Internet finance, digital finance, Fintech, financial technology, quantitative finance, open banking
