# Supplementary material for: Enterprise digital transformation’s impact on stock liquidity: A corporate governance perspective
Source: PLoS One. 2024 Mar 20;19(3):e0293818. doi: 10.1371/journal.pone.0293818 (PMC10954117; doi:10.1371/journal.pone.0293818)
Supplement: Attachment — Submitted filename: Response to reviewers.docx [file pone.0293818.s002.docx]

****Response to reviewers****

Dear reviewer,

Thank you very much for your comients and professional advice. These opinions help to improve academic rigor of our study, we have mad ecorrected modifications on the revised manuscript. Meanwlile, We proofread and revise the text, grammar, and punctuation of the manuscript. We hope that our work can be improved again. Furthermore, we would like to show the details as follows:

**Reviewer #1**

**1.** I would like to ask you to improve the literature review section of your manuscript by incorporating relevant papers. Specifically, we suggest that you consider the following papers:

**1.1** Bao and Huang (2021) "Shadow banking in a crisis: Evidence from FinTech during COVID-19" and Chen et al. (2022) "Dynamic correlation of market connectivity, risk spillover and abnormal volatility in stock price" for their insights on the impact of FinTech on financial markets and stock price dynamics.

**The author's answer:**

We sincerely thank you for your valuable comments.We have carefully read the two articles by Bao and Huang (2021) and Chen et al. (2022) that you suggested.As you said, the conclusions of the financial technology-related research in the paper "Shadow banking in a crisis: Evidence from FinTech during COVID-19" can improve the theoretical basis for our research on financial technology-related issues in the heterogeneity test, and add references to "6.1 Financial technology level" in the revised manuscript as references;the factors affecting abnormal fluctuations in stock prices in the paper "Dynamic correlation of market connectivity, risk spillover and abnormal volatility in stock price" can strengthen the theoretical basis for our research on stock price crash risk, and add references to "7 Further analysis" in the revised manuscript as references.

**1.2** Huang et al. (2022) "Semiparametric Single-Index Estimation for Average Treatment Effects" for its methodological contribution to causal inference, which may be relevant to your analysis of the impact of enterprise digital transformation on stock liquidity.

**The author's answer:**

We sincerely thank you for your valuable comments.We have carefully read your suggestion on Huang et al. (2022) "Semiparametric Single-Index Estimation for Average Treatment Effects".As you said, after reading this article, we found that the PSM research results involved in this article can effectively improve the theoretical foundation of the PSM method used in our study for endogenous test, and we have added a reference to the "4.4.2 Missing variables problem" in the revised manuscript.

**1.3** Yu et al. (2023a) "Cross-sectional uncertainty and expected stock returns" and Yu et al. (2023b) "Option-Implied Idiosyncratic Skewness and Expected Returns: Mind the Long Run" for their insights on stock return predictability, which may be relevant to your analysis of the economic consequences of enterprise digital transformation on stock liquidity.

**The author's answer:**

We sincerely thank you for your valuable comments.We have carefully read the two papers you suggested, Yu et al. (2023a) and Yu et al. (2023b).As you said, we found through reading that the research conclusion on stock return prediction involved in the article "Cross-sectional uncertainty and expected stock returns" can strengthen the theoretical foundation of our research on further analysis of analyst forecasting related research, and added a reference to the "7 Further analysis" in the revised manuscript.

After incorporating the papers you suggested into our literature review, we found that these papers can effectively strengthen the theoretical foundation of our research and provide a more concise and comprehensive explanation of the impact of corporate digital transformation on stock liquidity.

2. we would like to provide some comments to help improve your manuscript.

**2.1** we suggest that you provide more details on the data and methodology used in your study, including the sample selection criteria, variable definitions, and econometric models. This will help readers to better understand the empirical analysis and replicate your results.

**The author's answer:**

We greatly appreciate your professional input into our research.As you noted, we have provided more detailed information on the data and methods used in our research in the revised manuscript, as you requested.

(1) We have added detailed information on the data and methods used in the study in the revised manuscript "3.1 Data and sample".We have added more information on the reasons for selecting the study sample period, the measurement tools used in the study, and the source of the study sample data.As you may be concerned, adding these details to the study can help readers better understand the reasons for selecting the sample.

(2) In the "3.2.1 Explained variable: stock liquidity" section of the revised manuscript, we have added more information about the definition and measurement process of stock liquidity variables, as well as the reasons and theoretical basis for selecting the Amihud research method as the measurement model.As you may be concerned, supplementing these contents in the study can help readers better understand and replicate the stock liquidity variable, and also strengthen the theoretical basis of the explained variable in the study.

(3) In the "3.2.2 Explanatory variable: digital transformation" section of the revised manuscript, we have added a more complete definition and measurement process for enterprise digital transformation, as well as the reasons and theoretical basis for selecting text analysis as a measurement method for enterprise digital transformation. In the appendix, we have also added a keyword library of enterprise digital transformation used in the study.As you may be aware, the inclusion of these additional details in the study will help readers better understand and replicate the variable of enterprise digital transformation, as well as strengthen the theoretical basis and logical representation of the explanatory variables in the study.

(4) In the "3.2.3 Control variables" section of the revised manuscript, we have added the basis for selecting control variables in the study. As you may be concerned, the inclusion of these details in the study will help readers better understand the reasons and basis for selecting control variables.

(5) In the revised manuscript, we have modified the reasons for setting the benchmark regression model, the theoretical basis for model construction, and the econometric tools used for empirical testing in "3.3 Model design". As you may be concerned, adding these elements to the study can help readers better understand the testing model set in the study.

(6) In the "4.3 Robustness test" section of the revised manuscript, we have added the reasons for choosing the replacement variable as the robustness test method in the study.In the "4.3.1 Replace the explained variable" section, we have added the purpose of testing the replacement of the explained variable and the theoretical basis for selecting the replacement variable.In the "4.3.2 Replace the explanatory variables" section, we have added the purpose of testing the replacement of the core explanatory variable, the definition of the replacement variable, and the theoretical basis for selecting the replacement variable.As you may be concerned, the supplementary explanation of the replacement variable involved in the robustness test in the study will help readers better understand the method used in the robustness test in this study.

(7) In the "4.4 Endogeneity test" section of the revised manuscript, we have added information on the endogenous test method.In the "4.4.1 Reverse causation problem" section, we have added information on the use of lagged core explanatory variables and the two-stage least squares method with instrumental variables to address the causes, theoretical basis, and testing objectives of the endogenous problem caused by omitted variables.In the "4.4.2 Missing variables problem" section, we have added information on the reasons, objectives, theoretical basis, references, and sentence expression of using PSM and multi-period DID methods to address the endogenous problem.As you may be concerned, the addition of relevant information on the endogenous test in the study can help readers better understand the purpose and significance of the methods used in the endogenous test in this study.

(8) In the "5. Mechanism analysis" section of the revised manuscript, we have added the theoretical basis and testing objectives of the stepwise testing method used for mechanism testing. As you have noted, the addition of relevant information in the stepwise testing method in the study can help readers better understand the model used in this study to test the mediating variables of financing constraints, internal control, and information disclosure, and also increases the theoretical basis for mechanism testing.

(9) In the "6 Heterogeneity analysis" section of the revised manuscript, we have added three criteria for grouping heterogeneity tests: fintech, market development level, and digital policy guidance.As you may be aware, the inclusion of grouping criteria in the study can help readers better understand the process and results of heterogeneity tests.

(10) In the "7 Further analysis" section of the revised manuscript, we have added theoretical foundations for two indicators: stock price crash risk and analyst forecast quality. As you may be aware, adding theoretical foundations to research can help readers better understand the reasons and methods for selecting economic consequences indicators.

**2.2** we suggest that you provide more discussion on the limitations of your study and avenues for future research. Specifically, you may consider discussing the potential endogeneity issues in your analysis and exploring the impact of enterprise digital transformation on other aspects of firm performance, such as profitability and innovation.

**The author's answer:**

We greatly appreciate your professional advice on the limitations of our research and future research avenues.As you noted, we have added the limitations of the study and future research directions in the "8 Conclusions and recommendations" section of the revised manuscript.

(1) We believe that there are limitations in the scope of application of our research conclusions, which are limited to the economic effects of enterprise digital transformation on the capital market, and cannot reflect the full range of economic effects of enterprise digital transformation.As you have suggested, in our future research, we should focus on the economic effects of enterprise digital transformation on other aspects of enterprise performance, such as profitability and innovation performance.

(2) We believe that we cannot completely eliminate endogenous problems in our endogenous test.As you suggested, we should add more endogenous test models and methods, such as the Heckman two-stage model and GMM estimation method, in our future research.

(3) We believe that selecting Chinese listed companies from 2012 to 2021 as the research sample in this study is restrictive.As you have noted, in future research we need to add sample data from other countries or regions for research.

**Reviewer #2**

**1.** Introduction:
**1.1** The introduction should provide a clear and concise overview of the research topic, highlighting the significance and relevance of studying the impact of enterprise digital transformation on stock liquidity.

**The author's answer:**

We greatly appreciate your professional advice on our research.As you may be aware, in order to provide a clearer and more concise overview of the research topic in our revised manuscript, we have integrated the first two paragraphs of the introduction into the first paragraph of the revised manuscript, making the research subject more clearly and concisely described.At the same time, we have revised the relevant statements about the significance and relevance of the study on the impact of enterprise digital transformation on stock liquidity in the first paragraph of the revised manuscript, making the significance and relevance of the study more prominent and clearly described, helping readers better understand the research topic of this study.

**1.2** The introduction should also provide a brief literature review, discussing the existing research on this topic and identifying the research gap that your study aims to fill.

**The author's answer:**

We greatly appreciate your professional advice on our research.As you may be aware, we have restructured the original literature review in the first paragraph of the "1Introduction" section of our revised manuscript to highlight a concise discussion of the current research status of the research topic.At the same time, we have added a statement at the end of the third paragraph of the "1Introduction" section to describe the research gap that this study aims to fill, namely revealing the "mechanism black box" of the impact of enterprise digital transformation on stock liquidity. This fills the research gap in the micro-mechanism of the research topic, making the research purpose and research gap clear and explicit.

**1.3** Please ensure that the research objectives and research questions are clearly stated at the end of the introduction.

**The author's answer:**

We greatly appreciate your professional advice on our research.As you may be aware, in order to ensure that the research objectives and research questions are reflected at the end of the introduction, we have revised and added relevant statements about the research objectives and research questions in the final paragraph of the "1 Introduction" section of the revised manuscript, making the presentation of the research questions and research objectives in this study more prominent and clear.

**2.** Methodology:
**2.1** Provide a detailed description of the data sources and sample selection process. It is important to justify the choice of Chinese A-share listed companies from 2012-2021 as the dataset for this study.

**The author's answer:**

We greatly appreciate your professional input on our research.As you noted, we have added a description of the data sources and sample selection reasons used in this study in "3.1 Data and sample" of the revised manuscript, and have also included more detailed data descriptions and explanations in the attached files.

**2.2** Clearly explain the variables used to measure enterprise digital transformation and stock liquidity. Provide references for the measurement methods and discuss their validity and reliability.

**The author's answer:**

We greatly appreciate your professional advice on our research.As you may be aware, we have revised the statement on the measurement of variables measuring corporate digital transformation and stock liquidity in "3.2 Variable setting and description" in the revised manuscript, added references to the measurement methods, and discussed the validity of the methods.

(1) We have added more descriptions of stock liquidity measurement methods and measurement processes in "3.2.1 Explained variable: stock liquidity" in the revised manuscript, clarifying the variable measurement methods for measuring stock liquidity.We have also added the research results of scholars Chiang and Zheng and Le to support and discuss the validity and reliability of the research results used in the study to measure stock liquidity using Amihud and Mendelson's research results.

(2) We have added the detailed process of measuring the variable of enterprise digital transformation in "3.2.2 Explanatory variable: digital transformation" in the revised manuscript, clarified the text analysis method used in enterprise digital transformation, and supported and discussed the effectiveness and reliability of using text analysis to measure enterprise digital transformation, using the research results of scholars such as Jiang and Fang as references.

**2.3** Describe the statistical methods used for data analysis, including any econometric models or techniques employed. Justify the choice of these methods and discuss their appropriateness for addressing the research questions.

**The author's answer:**

We greatly appreciate your professional advice on our research.As you may be aware, we have made modifications to the sections involving data analysis methods and econometric models in the revised manuscript, and discussed the appropriateness of method selection for the research question.

(1) In the "3.1 Data and sample" section of the revised manuscript, we have added the main econometric tool used in this article, namely Stata17.0 software.

(2) In the "3.3 Model design" section of the revised manuscript, we provide a more detailed description of the econometric model used in this article's benchmark regression model, and discuss the purpose and applicability of using a multiple regression model as the benchmark regression model.

(3) In the "4.3 Robustness test" section of the revised manuscript, we have added a statement on the purpose and applicability of the test method used for robustness testing.Specifically, in the "4.3.1 Replace the explained variable" section, we have added the research conclusions of Ding and Hou, which verify the applicability and reliability of using the Zeros index and Roll index to replace the Amihud index to measure stock liquidity in the study to address the robustness issues of the benchmark regression model.

(4)In "4.3.2 Replace the explanatory variables" of the revised manuscript, we added the applicability and purpose of using the Wu et al. (2021) digital feature word library construction method and the digital feature word proportion measurement method to replace the Zhao (2021) enterprise digital transformation measurement method in our research, to verify the applicability and reliability of these two methods in solving the robustness problem of the benchmark regression model.

(5) We have added a discussion on the applicability of methods for addressing endogeneity issues in "4.4 Endogeneity test" in the revised manuscript.

Among them, "4.4.1 Reverse causation problem" supplements the reasons and purposes of using the lagged explanatory variables for periods 1-3 and the instrumental variable method in this study, and adds the research conclusions of Yonghong and Peng et al. to discuss the applicability and reliability of using this testing method to solve the endogenous problems that may exist in the benchmark regression model caused by reverse causation.

In "4.4.2 Missing variables problem", the purpose and reasons for using the multi-period DID model and PSM method to address the endogenous problem caused by omitted variables are added.The research results of Shipman, Ren et al. and other scholars are also added to discuss the applicability and reliability of using these two testing methods to solve the endogenous problem caused by omitted variables that may exist in the benchmark regression model.

(6) In the "5 Mechanism analysis" section of the revised manuscript, we have added more information on the mediating effect model used in this study, namely the stepwise approach, as well as research findings from scholars such as Baron and Kenny and Went et al., to support and discuss the applicability and reliability of using the causal steps approach to verify the impact mechanism of enterprise digital transformation on stock liquidity.

(7) In the "6 Heterogeneity analysis" section of the revised manuscript, we added three criteria for grouping heterogeneity tests: fintech, market development level, and digital policy guidance, which improved the applicability and reliability of using grouping tests to study subject heterogeneity.

(8) We have added the research conclusions of Chen, Yu, and other scholars in the "7 Further analysis" section of the revised manuscript to verify and discuss the applicability and reliability of using stock price crash risk and analyst forecast quality as indicators for economic consequences research.

**3.** Results:
**3.1** Present the results of the analysis in a clear and organized manner. Use tables and figures to effectively summarize and present the findings.

**The author's answer:**

We greatly appreciate your professional input on our research.As you may be aware, we have restructured the presentation of our research findings in the "8 Conclusions and recommendations" section of the revised manuscript, reclassifying and ordering the research conclusions to make them clearer and more logically presented.

**3.2** Clearly state the main findings of the study and discuss their implications. Relate the findings back to the research objectives and research questions stated in the introduction.

**The author's answer:**

We greatly appreciate your professional advice on our research.As you may be aware, we have revised the conclusions section of the "8 Conclusions and recommendations" in the revised manuscript to address the impact, mechanism, heterogeneity testing, and economic consequences of the research topic. This has strengthened the connection between the research conclusions and the objectives and research findings in the introduction, and fully discusses the economic significance and implications of the research conclusions.

**3.3** Provide a thorough discussion of the heterogeneity analysis results, highlighting the differences in the impact of enterprise digital transformation on stock liquidity in high-fintech areas, developed financial markets, and policy-guided enterprises.

**The author's answer:**

We greatly appreciate your professional advice on our research.As you may be aware, we have revised the statement of heterogeneity analysis conclusions in the "8 Conclusions and recommendations" section of the revised manuscript. By comparing the research conclusions of the two subgroups of fintech, financial market development, and policy guidance, we highlight the differences in the economic impact of enterprise digital transformation on stock liquidity under different external environments.

**4.** Discussion:
**4.1** The discussion section should provide a comprehensive interpretation and analysis of the results, relating them to the existing literature and theoretical frameworks.

**The author's answer:**

We greatly appreciate your professional advice on our research.As you have noted, we have revised the discussion section of the "8 Conclusions and recommendations" in the revised manuscript. We have re-interpreted and analyzed the research conclusions on the impact, mechanism, heterogeneity analysis, and economic consequences of enterprise digital transformation on stock liquidity from the government and enterprise levels. We have provided practical suggestions based on the research conclusions, and added the research conclusions of Li and Luo, Zhao, Agarwal, Lai, and other scholars in the interpretation process, combined with the theoretical framework of the previous article, making the discussion and suggestions on the research conclusions more practical and theoretical.

**4.2** Discuss the mechanisms through which enterprise digital transformation improves stock liquidity, specifically addressing the three mechanisms identified in the study: easing financing constraints, enhancing internal control quality, and improving information disclosure levels.

**The author's answer:**

We greatly appreciate your professional advice on our research.As you may be aware, we have revised the mechanism discussion section in the "8 Conclusions and recommendations" section of the revised manuscript. We have made suggestions on the practical path of improving stock liquidity for enterprises' digital transformation from the perspectives of financing constraints, internal control, and information disclosure at the levels of governments and enterprises. We have also cited the research conclusions of Li and Luo, Zhao, Agarwal, and other scholars, as well as the theoretical framework presented earlier, to ensure the reliability and applicability of the practical suggestions proposed in this study.

**4.3** Discuss the economic consequences of enterprise digital transformation on stock liquidity, focusing on the reduction of stock price crashes and the improvement of analysts' forecasts.

**The author's answer:**

We greatly appreciate your professional advice on our research.As you may have noticed, we have revised the discussion of the economic consequences in the "8 Conclusions and recommendations" section of the revised manuscript.We discuss the economic consequences of corporate digital transformation on stock liquidity from the perspectives of governments and enterprises, and specifically propose concrete measures to reduce the risk of stock price collapse and improve the quality of analyst forecasts. We introduce the research conclusions of scholars such as Chauhan and Chen and the theoretical framework described earlier, ensuring the reliability and applicability of the practical recommendations on economic consequences proposed in this study.

**4.5 Clearly state the limitations of the study and suggest avenues for future research.**

**The author's answer:**

We greatly appreciate your professional advice on the limitations of our research and future research avenues.As you noted, we have added the limitations of this study and future research directions in the "8 Conclusions and recommendations" section of the revised manuscript.

(1) We believe that there are limitations in the scope of application of our research conclusions, which are limited to the economic effects of enterprise digital transformation on the capital market, and cannot reflect the full range of economic effects of enterprise digital transformation.We propose that in future research, we should focus on the economic effects of enterprise digital transformation on other aspects of enterprise performance, such as profitability and innovation performance.

(2) We believe that we cannot fully eliminate endogenous problems in our endogenous test.We propose that we should add more endogenous testing models and methods, such as the Heckman two-stage model and the GMM estimation method, in future research.

(3) We believe that selecting Chinese listed companies from 2012 to 2021 as the research sample in this study is restrictive.We propose that in future research, we need to add sample data from other countries or regions for research.

**5.** Abstract:
The abstract should be revised to accurately reflect the content and findings of the study. It should provide a clear and concise summary of the research objectives, methodology, results, and implications.

**The author's answer:**

We greatly appreciate your professional feedback on the summary of our research.As you noted, we have revised the abstract in the revised manuscript to optimize the order of language presentation, and have re-summarized the research content and results of this study to ensure that the summary clearly and accurately reflects the research objectives, methods, and results of this study.

**6.** Writing style and clarity:
**6.1** The manuscript should be carefully proofread for grammar, spelling, and punctuation errors.

**The author's answer:**

We greatly appreciate your professional feedback on the summary of our research.As you noted, we have carefully reviewed the manuscript and made corrections to grammatical, spelling, and punctuation errors that occurred during the research.

**6.2** Ensure that the writing is clear, concise, and well-organized. Use headings and subheadings to structure the manuscript and guide the reader through the content.

**The author's answer:**

We greatly appreciate your professional feedback on the summary of our research.As you noted, we have revised some of the textual expressions in the revised manuscript and added subheadings where appropriate, such as adding a subheading for robustness testing in "4.4 Endogeneity test", making the textual expression of this study clearer and more concise, making it easier for readers to understand the content of this study.

**6.3** Avoid excessive jargon and technical terms. Define any specialized terms or concepts that may be unfamiliar to a general audience.

**The author's answer:**

We greatly appreciate your professional comments on the summary of our research.As you may be aware, we often use colloquial expressions in our research to convey the content, and we try to choose nouns that are understandable to general readers for variable names.

**Modification description of references**

In order to express the theoretical framework more clearly and concisely and enhance the theoretical foundation of this article, we have made modifications to the references in the revised manuscript.

**1. The following references have been added:**

Chiang T C, Zheng D. Liquidity and stock returns: Evidence from international markets[J]. Global Finance Journal, 2015, 27: 73-97. [https://doi.org/10.1016/j.gfj.2015.04.005](https://doi.org/10.1016/j.gfj.2015.04.005)

Kumari J. Investor sentiment and stock market liquidity: Evidence from an emerging economy. Journal of Behavioral and Experimental Finance. 2019;23:166-80. https://doi.org/10.1016/j.jbef.2019.07.002

Ding R, Hou W. Retail investor attention and stock liquidity. Journal of international financial markets, institutions and money. 2015;37:12-26. https://doi.org/10.1016/j.intfin.2015.04.001

Yonghong L, Jie S, Ge Z, Ru Z. The impact of enterprise digital transformation on financial performance—Evidence from Mainland China manufacturing firms. Managerial and Decision Economics. 2023;44(4):2110-24. https://doi.org/10.1002/mde.3805

Shipman JE, Swanquist QT, Whited RL. Propensity score matching in accounting research. The Accounting Review. 2017;92(1):213-44. https://doi.org/10.2308/accr-51449

Huang D, Gao J, Oka T. Semiparametric Single-Index Estimation for Average Treatment Effects. arXiv preprint arXiv:220608503. 2022. https://doi.org/10.48550/arXiv.2206.08503

Ren X, Zeng G, Zhao Y. Digital finance and corporate ESG performance: Empirical evidence from listed companies in China. Pacific-Basin Finance Journal. 2023;79:102019.

Bao Z, Huang D. Shadow banking in a crisis: Evidence from FinTech during COVID-19. Journal of Financial and Quantitative Analysis. 2021;56(7):2320-55. https://doi.org/10.1017/S0022109021000430

Chen M, Li N, Zheng L, Huang D, Wu B. Dynamic correlation of market connectivity, risk spillover and abnormal volatility in stock price. Physica A: Statistical Mechanics and Its Applications. 2022;587:126506. [https://doi.org/10.1016/j.physa.2021.126506](https://doi.org/10.1016/j.physa.2021.126506)

Yu D, Huang D. Cross-sectional uncertainty and expected stock returns. Journal of Empirical Finance. 2023;72:321-40. [https://doi.org/10.1016/j.jempfin.2023.04.001](https://doi.org/10.1016/j.jempfin.2023.04.001)

Yu D, Huang D. Option-Implied Idiosyncratic Skewness and Expected Returns: Mind the Long Run. Available at SSRN 4323748. 2023. https://ssrn.com/abstract=4323748

Li X, Luo D. Financial constraints, stock liquidity, and stock returns. Journal of International Financial Markets, Institutions and Money. 2019;63:101139. https://doi.org/10.1016/j.intfin.2019.101139

Zhao T, Yan N, Ji L. Digital transformation, life cycle and internal control effectiveness: Evidence from China. Finance Research Letters. 2023;58:104223. https://doi.org/10.1016/j.frl.2023.104223

Agarwal V, Mullally KA, Tang Y, Yang B. Mandatory portfolio disclosure, stock liquidity, and mutual fund performance. The Journal of Finance. 2015;70(6):2733-76. <https://doi.org/10.1111/jofi.12245>

Lee C-H, Chou P-I. Financial openness and market liquidity in emerging markets. Finance Research Letters. 2018;25:124-30. https://doi.org/10.1016/j.frl.2017.10.024

Zaremba A, Aharon DY, Demir E, Kizys R, Zawadka D. COVID-19, government policy responses, and stock market liquidity around the world: A note. Research in International Business and Finance. 2021;56:101359. [https://doi.org/10.1016/j.ribaf.2020.101359](https://doi.org/10.1016/j.ribaf.2020.101359)

Chen W, Zhang L, Jiang P, Meng F, Sun Q. Can digital transformation improve the information environment of the capital market? Evidence from the analysts' prediction behaviour. Accounting & Finance. 2022;62(2):2543-78. https://doi.org/10.1111/acfi.12873

**2. The following references were deleted:**

Ren C, Lee S J, Hu C. Digitalization Improves Enterprise Performance: New Evidence by Text Analysis[J]. SAGE Open, 2023, 13(2): 21582440231175871.doi:https://doi.org/10.1177/21582440231175871

Le H, Gregoriou A. How do you capture liquidity? A review of the literature on low‐frequency stock liquidity. Journal of Economic Surveys. 2020;34(5):1170-86.

Dey A. Corporate governance and agency conflicts. Journal of accounting research. 2008;46(5):1143-81. <https://doi.org/10.1111/j.1475-679X.2008.00301.x>

Wang K, Li T, San Z, Gao H. How does corporate ESG performance affect stock liquidity? Evidence from China. Pacific-Basin Finance Journal. 2023;80:102087. https://doi.org/10.1016/j.pacfin.2023.102087

Jiang F, Ma Y, Shi B. Stock liquidity and dividend payouts. Journal of Corporate finance. 2017;42:295-314. https://doi.org/10.1016/j.jcorpfin.2016.12.005

Xue L, Zhang C, Ling H, Zhao X. Risk mitigation in supply chain digitization: System modularity and information technology governance. Journal of Management Information Systems. 2013;30(1):325-52. https://doi.org/10.2753/MIS0742-1222300110

Zhang F, Yang B, Zhu L. Digital technology usage, strategic flexibility, and business model innovation in traditional manufacturing firms: The moderating role of the institutional environment. Technological Forecasting and Social Change. 2023;194:122726. https://doi.org/10.1016/j.techfore.2023.122726

Zhong Y, Zhao H, Yin T. Resource Bundling: How Does Enterprise Digital Transformation Affect Enterprise ESG Development? Sustainability. 2023;15(2):1319. https://doi.org/10.3390/su15021319

Gao D, Yan Z, Zhou X, Mo X. Smarter and Prosperous: Digital Transformation and Enterprise Performance. Systems. 2023;11(7):329. https://doi.org/10.3390/systems11070329

Wang Z. Digital finance, financing constraint and enterprise financial riskJ]. Journal of Mathematics, 2022, 2022. https://doi.org/10.1155/2022/2882113

Thank you very much for your attention and time. Look forward to hearing from you.

Yours sincerely

Jia Zhu

Oct 11, 2023

Zhengzhou University of Aeronautics
