# Supplementary material for: Enterprise digital transformation’s impact on stock liquidity: A corporate governance perspective
Source: PLoS One. 2024 Mar 20;19(3):e0293818. doi: 10.1371/journal.pone.0293818 (PMC10954117; doi:10.1371/journal.pone.0293818)
Supplement: Attachment — Submitted filename: Response to reviewers.docx [file pone.0293818.s003.docx]

**Response to reviewers**

Dear reviewer,

Thank you very much for your comients and professional advice. These opinions help to improve academic rigor of our study, we have mad ecorrected modifications on the revised manuscript. Meanwlile, We proofread and revise the text, grammar, and punctuation of the manuscript. We hope that our work can be improved again. Furthermore, we would like to show the details as follows:

**Reviewer #1**

**1.** To enhance the literature review, we suggest that you consider the following papers:

**1.1** Huang, D., Li, Y., Wang, X., & Zhong, Z. (2022). Does the Federal Open Market Committee cycle affect credit risk? Financial Management, 51(1), 143–167.

**The author's answer:**

We sincerely thank you for your valuable comments.We have carefully read your suggestion of "Does the Federal Open Market Committee cycle affect credit risk?", and found that the research conclusion in this article on the impact of the transmission of external information signals on stock risk premiums can enhance the theoretical foundation of our research on information disclosure. We have added this article as a reference in "2.2.3 Digital transformation, information disclosure, and stock liquidity".

**1.2** Huang, D., Wang, X., & Zhong, Z. (2020). Monetary Policy Surprises and Corporate Credit Spreads. Available at SSRN 3700257.

**The author's answer:**

We sincerely thank you for your valuable comments.We have carefully read your suggested article "Monetary Policy Surprises and Corporate Credit Spreads" and found that the research conclusions on the impact of monetary policy and other financial market conditions on corporate credit spreads in this article can enhance the theoretical foundation of our research on financial market-related heterogeneity analysis. We have added this article as a reference in the section "6.2 Degree of financial market development" for further reference.

**1.3** Li, N., Chen, M., Gao, H., Huang, D., & Yang, X. (2023). Impact of lockdown and government subsidies on rural households at early COVID-19 pandemic in China. China Agricultural Economic Review, 15(1), 109–133.

**The author's answer:**

We sincerely thank you for your valuable comments.We have carefully read your suggested "Impact of lockdown and government subsidies on rural households at early COVID-19 pandemic in China", and found that the research on government subsidies in this article can enhance the theoretical foundation of our research on government policy formulation in our conclusions and recommendations. We have added this article as a reference in the "8 Conclusions and recommendations".

**1.4** Li, N., Chen, M., & Huang, D. (2022). How Do Logistics Disruptions Affect Rural Households? Evidence from COVID-19 in China. Sustainability, 15(1), 465.

**The author's answer:**

We sincerely thank you for your valuable comments.We have carefully read your suggested article "How Do Logistics Disruptions Affect Rural Households? Evidence from COVID-19 in China." and found that the research conclusions involving supply chain management in this article can enhance the theoretical basis for our conclusions and recommendations for government policy formulation and enterprise supply chain management standards. We have added this article as a reference in the "8 Conclusions and recommendations" section.

**1.5** Wu, B., Huang, D., & Chen, M. (2023). Estimating contagion mechanism in global equity market with time-zone effect. Financial Management, 52, 543–572.

**The author's answer:**

We sincerely thank you for your valuable comments.We have carefully read your suggested "Estimating contagion mechanism in global equity market with time-zone effect" and found that this article validates the connectivity of global stock markets, which can enhance the theoretical basis for governments in our conclusions and recommendations to exchange and formulate unified policies. We will add this article as a reference in the "8 Conclusions and recommendations".

**1.6** Yu, D., Huang, D., & Chen, L. (2023). Stock return predictability and cyclical movements in valuation ratios. Journal of Empirical Finance, 72, 36–53.

**The author's answer:**

We sincerely thank you for your valuable comments.We have carefully read your suggested "Stock return predictability and cyclical movements in valuation ratios" and found that the research conclusions related to the predictability of stock returns in this article can enhance the theoretical foundation for our further research on the quality of analyst forecasts. We will add this article as a reference in the "7 Further analysis" section.

**1.7** Yu, D., Huang, D., Chen, L., & Li, L. (2023). Forecasting dividend growth: The role of adjusted earnings yield. Economic Modelling, 120, 106188.

**The author's answer:**

We sincerely thank you for your valuable comments.We have carefully read your suggested "Forecasting dividend growth: The role of adjusted earnings yield" and found that the research conclusions related to the factors affecting dividend forecasts in this article can enhance the theoretical basis for improving the quality of analysts' forecasts in our research conclusions and recommendations. We will add this article as a reference in the "8 Conclusions and recommendations" section.

**1.8** Zhang, Y., Huang, D., Xiang, Z., Yang, Y., & Wang, X. (2023). Expressed Sentiment on Social Media During the COVID-19 Pandemic: Evidence from the Lockdown in Shanghai. Available at SSRN 4486863.

**The author's answer:**

We sincerely thank you for your valuable comments.We have carefully read your suggested "Expressed Sentiment on Social Media During the COVID-19 Pandemic: Evidence from the Lockdown in Shanghai" and found that the research conclusions related to the impact of social media information dissemination in this article can enhance the theoretical basis for our research conclusions and recommendations for the government to improve the quality of information disclosure. We will add this article as a reference in the "8 Conclusions and recommendations" section.

**1.9** Zhou, Y., Huang, D., Chen, M., Wang, Y., & Yang, X. (2022). How Did Small Business Respond to Unexpected Shocks? Evidence from a Natural Experiment in China.

**The author's answer:**

We sincerely thank you for your valuable comments.We have carefully read your suggested "How Did Small Business Respond to Unexpected Shocks? Evidence from a Natural Experiment in China" and found that the research conclusions related to enterprises' response to external shocks in this article can enhance the theoretical basis for our research conclusions and suggestions for enterprises to improve their internal control quality. We have added this article as a reference in the "8 Conclusions and recommendations" section.

**2.** In addition to improving the literature review, we have the following comments to help you improve the submission:

**2.1** Please provide a more detailed explanation of the methodology used in your study, including the data sources, sample selection criteria, and the econometric models employed. This will help readers better understand the robustness of your findings.

**The author's answer:**

(1)In Revised manuscript "3.1Data and sample", we explained the sample selection criteria in more detail, as well as the reasons for selecting Chinese listed companies from 2012 to 2021 as the research sample.In the study, we also provided a more detailed description of the data sources, and explained the data sources and calculation methods in the accompanying documents.

(2)In Revised manuscript "3.3 Model design", we explained in more detail the reasons for selecting the multiple linear regression model as the main econometric model for this study, and explained the testing logic of the multiple linear regression model.

(3)In Revised manuscript "4.4.1Reverse causation problem", we explain in more detail the reasons for using instrumental variables as an endogenous test method in this study, explaining the logic and role of instrumental variables in the econometric process.

(4)In Revised manuscript "4.4.2Missing variables problem", we explained in more detail the reasons for using the PSM model to address the endogeneity issues caused by omitted variables in this study, and explained the logic and role of the PSM method in the econometric process.

(5)In Revised manuscript "4.4.2Missing variables problem", we explained in more detail the reasons for using a multi-period DID model to address the endogeneity problem caused by omitted variables in this study, and explained the logic and role of the multi-period DID model in the econometric process.

(6) In Revised manuscript "5 Mechanism analysis", we explain in more detail the reasons for using the stepwise testing method to verify the mechanism of the impact of enterprise digital transformation on stock liquidity in this study, and supplement the advantages and testing logic of the stepwise testing method in mediating tests.

**2.2** Consider discussing the potential endogeneity issues that may arise in your analysis, such as reverse causality or omitted variable bias. If possible, address these concerns by employing appropriate econometric techniques, such as instrumental variable estimation or propensity score matching.

**The author's answer:**

We greatly appreciate your professional advice on our research.As you have noted, we have carefully analyzed the endogenous issues of the impact of enterprise digital transformation on stock price crash risk and analyst forecast quality in accordance with what you said in Revised manuscript "7Further analysis". In our research, we found that instrumental variables have certain advantages in dealing with endogenous issues caused by omitted variables, reverse causality, sample selection, and measurement error. Therefore, we chose the instrumental variable method in Revised manuscript "7Further analysis" to deal with potential endogenous issues.

**2.3** Elaborate on the policy implications of your findings. How can policymakers and regulators use the insights from your study to promote enterprise digital transformation and enhance stock liquidity in the capital market?

**The author's answer:**

We greatly appreciate your professional advice on our research.As you have noted, we have reflected more deeply on the practical significance of our research findings for governments and regulatory agencies in Revised manuscript "8 Conclusions and recommendations", and have added suggestions for policy makers and regulators on practical paths to promote enterprise digital transformation and improve stock liquidity in the capital market.

**2.4** Provide a more in-depth discussion of the limitations of your study and potential avenues for future research. This will help readers better understand the generalizability of your findings and identify areas where further investigation is needed.

**The author's answer:**

We greatly appreciate your professional advice on our research.As you have noted, we have discussed in greater depth the research limitations, causes, and solutions of our research in Revised manuscript "8 Conclusions and recommendations" in accordance with your suggestions. We have also provided detailed supplementary information on the specific methods and implementation paths used to compensate for the limitations in future research.

**Reviewer #3:**

**1.** I commend the authors for the substantive improvements made to the paper after the initial round of feedback. However, I still believe there's an opportunity to strengthen the introduction and motivation sections. It would be beneficial if the authors commenced their motivation with a foundational discourse on technology, encompassing areas like blockchain, AI, and their subsequent impact on corporate outcomes. I recommend starting this discussion by referencing the seminal work by An & Rau (2021) titled "Finance, technology and disruption. The European Journal of Finance, 27(4-5), 334-345."This paper offers a robust theoretical framework elucidating technology's influence on the corporate realm. Following this, a brief one or two sentences on the overarching advantages of technology within financial markets would be apt. The work by An et al. (2019), "Initial coin offerings and entrepreneurial finance: the role of founders’ characteristics. The Journal of Alternative Investments, 21(4), 26-40," serves as an excellent reference in this regard.To balance the narrative, it's crucial to also touch upon the potential drawbacks or challenges posed by technology in both the financial domain and the broader societal context. For this aspect, the study by An, Ding, and Lin (2023), "ChatGPT: tackling the growing carbon footprint of generative AI. Nature, 615(7953), 586-586," can be referenced as a pertinent example. By integrating these elements, the authors can effectively highlight the duality—both the promise and perils—of technological advancements.

**The author's answer:**

We greatly appreciate your professional advice on our research.As you have noted, we have revised and supplemented the research on the impact of digital technology on enterprises and financial markets in Revised manuscript "1Introduction" in accordance with your suggestions.We first elaborated on the impact of digital technology on enterprises and financial markets, followed by a discussion of the advantages of digital technology-driven changes in financial markets, and finally analyzed the advantages and challenges of technological progress for markets and enterprises.

At the same time, we sincerely thank you for recommending the references you provided. We have carefully read the three articles by An & Rau (2021)"Finance, technology and disruption"; An et al. (2019)"Initial coin offerings and entrepreneurial finance: the role of founders’ characteristics"; An, Ding, and Lin (2023)"ChatGPT: tackling the growing carbon footprint of generative AI".We found that the research results on the impact of technology on enterprises in An & Rau (2021) can effectively strengthen the theoretical foundation of the impact of technology on enterprises;the research conclusions on the impact of technology on financial markets in An et al. (2019) can provide reference for the overwhelming advantages of technology in financial markets;and the research conclusions on the potential disadvantages or challenges of technology in An, Ding, and Lin (2023) strengthen the theoretical foundation of the advantages and challenges brought by digital technology to enterprises.Therefore, we will cite these three articles as references in the revision of Revised manuscript "1Introduction".

**Modification description of references**

In order to express the theoretical framework more clearly and concisely and enhance the theoretical foundation of this article, we have made modifications to the references in the revised manuscript.

**1. The following references have been added:**

Huang D, Li Y, Wang X, Zhong Z. Does the Federal Open Market Committee cycle affect credit risk? Financial Management. 2022;51(1):143-67. <https://doi.org/10.1111/fima.12364>

Huang D, Wang X, Zhong Z. Monetary Policy Surprises and Corporate Credit Spreads. Available at SSRN 3700257. 2020. http://dx.doi.org/10.2139/ssrn.3700257

Li N, Chen M, Gao H, Huang D, Yang X. Impact of lockdown and government subsidies on rural households at early COVID-19 pandemic in China. China Agricultural Economic Review. 2023;15(1):109-33. [https://doi.org/10.1108/CAER-12-2021-0239](https://doi.org/10.1108/CAER-12-2021-0239)

Li N, Chen M, Huang D. How Do Logistics Disruptions Affect Rural Households? Evidence from COVID-19 in China. Sustainability. 2022;15(1):465. <https://doi.org/10.3390/su15010465>

Wu B, Chen M, Huang D. Estimating Contagion Mechanism in Global Equity Market with Time-Zone Effects. Available at SSRN 3491596. 2019. http://dx.doi.org/10.2139/ssrn.3491596

Yu D, Huang D, Chen L. Stock return predictability and cyclical movements in valuation ratios. Journal of Empirical Finance. 2023;72:36-53. [https://doi.org/10.1016/j.jempfin.2023.02.004](https://doi.org/10.1016/j.jempfin.2023.02.004)

Yu D, Huang D, Chen L, Li L. Forecasting dividend growth: The role of adjusted earnings yield. Economic Modelling. 2023;120:106188. [https://doi.org/10.1016/j.econmod.2022.106188](https://doi.org/10.1016/j.econmod.2022.106188)

Zhang Y, Huang D, Xiang Z, Yang Y, Wang X. Expressed Sentiment on Social Media During the COVID-19 Pandemic: Evidence from the Lockdown in Shanghai. Available at SSRN 4486863. 2023.http://dx.doi.org/10.2139/ssrn.4486863

Zhou Y, Huang D, Chen M, Wang Y, Yang X. How Did Small Business Respond to Unexpected Shocks? Evidence from a Natural Experiment in China. Evidence from a Natural Experiment in China (March 26, 2022). 2022. http://dx.doi.org/10.2139/ssrn.4044677

An J, Rau R. Finance, technology and disruption. The European Journal of Finance. 2021;27(4-5):334-45. https://doi.org/10.1080/1351847X.2019.1703024

An J, Duan T, Hou W, Xu X. Initial coin offerings and entrepreneurial finance: the role of founders’ characteristics. The Journal of Alternative Investments. 2019;21(4):26-40. https://doi.org/10.3905/jai.2019.1.068

An J, Ding W, Lin C. ChatGPT: tackle the growing carbon footprint of generative AI. Nature. 2023;615(7953):586-. https://doi.org/10.1038/d41586-023-00843-2

**2. The following references were deleted:**

Meng-tao C, Da-peng Y, Wei-qi Z, Qi-jun W. How does ESG disclosure improve stock liquidity for enterprises—Empirical evidence from China. Environmental Impact Assessment Review. 2023;98:106926. https://doi.org/10.1016/j.eiar.2022.106926

Sama LM, Stefanidis A, Casselman RM. Rethinking corporate governance in the digital economy: The role of stewardship. Business Horizons. 2022;65(5):535-46. https://doi.org/10.1016/j.bushor.2021.08.001

Fang VW, Noe TH, Tice S. Stock market liquidity and firm value. Journal of financial Economics. 2009;94(1):150-69. https://doi.org/10.1016/j.jfineco.2008.08.007

Karolyi GA, Lee K-H, Van Dijk MA. Understanding commonality in liquidity around the world. Journal of financial economics. 2012;105(1):82-112. https://doi.org/10.1016/j.jfineco.2011.12.008

Brogaard J, Li D, Xia Y. Stock liquidity and default riskJ]. Journal of Financial Economics, 2017, 124(3): 486-502. https://doi.org/10.1016/j.jfineco.2017.03.003

Li D, Shen W. Can corporate digitalization promote green innovation? The moderating roles of internal control and institutional ownership. Sustainability. 2021;13(24):13983. https://doi.org/10.3390/su132413983

Liu G, Liu B. How digital technology improves the high-quality development of enterprises and capital markets: A liquidity perspective. Finance Research Letters. 2023;53:103683. https://doi.org/10.1016/j.frl.2023.103683

Forcadell FJ, Aracil E, Úbeda F. The impact of corporate sustainability and digitalization on international banks’ performance. Global Policy. 2020;11:18-27. https://doi.org/10.1111/1758-5899.12761

He F, Qin S, Liu Y, Wu JG. CSR and idiosyncratic risk: Evidence from ESG information disclosure. Finance Research Letters. 2022;49:102936. https://doi.org/10.1016/j.frl.2022.102936

Mohammed A, de Sousa Jabbour ABL, Koh L, Hubbard N, Jabbour CJC, Al Ahmed T. The sourcing decision-making process in the era of digitalization: A new quantitative methodology. Transportation Research Part E: Logistics and Transportation Review. 2022;168:102948. https://doi.org/10.1016/j.tre.2022.102948

Sun Q, Shi Y. Will Digital Disclosure Affect Corporate Stocks?-Based on Stakeholders' Cognition. Engineering Economics. 2023;34(2):223-9. https://doi.org/10.5755/j01.ee.34.2.31514

**Data Provision Statement**

The data used in this study is provided in the supporting information in the form of an attachment.
