# Supplementary material for: Enterprise digital transformation’s impact on stock liquidity: A corporate governance perspective
Source: PLoS One. 2024 Mar 20;19(3):e0293818. doi: 10.1371/journal.pone.0293818 (PMC10954117; doi:10.1371/journal.pone.0293818)
Supplement: Attachment — Submitted filename: Response to reviewers.docx [file pone.0293818.s005.docx]

**Response to reviewers**

Dear reviewer,

Thank you very much for your comients and professional advice. These opinions help to improve academic rigor of our study, we have mad ecorrected modifications on the revised manuscript. Meanwlile, We proofread and revise the text, grammar, and punctuation of the manuscript. We hope that our work can be improved again. Furthermore, we would like to show the details as follows:

**Reviewer #1**

**1.** Please provide more information on the sample selection process, including any potential biases or limitations in the data.

**The author’s answer:**

We sincerely thank you for your valuable comments.In Revised manuscript “3.1 Data and sample”,We supplement the details of the selection process, including the digital transformation of enterprises, stock liquidity and the possible bias in control variables in the process of selection and calculation.

**2.** Consider discussing the implications of your findings for policymakers and practitioners, as well as any potential limitations or areas for future research.

**The author’s answer:**

We sincerely thank you for your valuable comments.In Revised manuscript “8 Conclusions and recommendations”,We supplement the impact of the results on policy makers and practitioners, and advise policy makers, business managers, and institutional investors in the context of the digital economy era.At the same time, we supplement the limitations of the enterprise digital transformation, the limitation of the stock liquidity and control variables affected in the selection and calculation process, and propose future research directions.

**Reviewer #2:**

**The author’s answer:**

We greatly appreciate your professional review of our study in such valuable time.

**Reviewer #3:**

The authors have addressed all my comments to a satisfactory level. I have no further comments.

**The author’s answer:**

We greatly appreciate your professional review of our study in such valuable time.

**Reviewer #4:**

**1.** The bid-ask spread is a commonly used proxy for stock liquidity, as employed by Fang et al. (2014) and Brogaard et al. (2017). It is advisable to enhance the robustness of the baseline results using bid-ask spread to measure stock liquidity.

**The author’s answer:**

We sincerely thank you for your valuable comments.In Revised manuscript “4.3.1Replace the explained variable”, We according to your suggestion by reading the references we to the difference between the selling price and purchase price and the ratio of stock liquidity, to replace the stock liquidity and benchmark regression test the robustness of the test results, the estimated coefficient of bid-ask spread in 1% of the confidence level is significantly negative, consistent with the benchmark regression results.

**2.** The DID model should be based on exogenous shocks, which are not determined by the research objectives, namely the firms in this paper. Constructing a quasi-natural experiment using enterprise digital transformation behavior may not be appropriate, as digital transformation is a company’s own development strategy. For instance, policies related to digital transformation could provide an ideal setting for the DID model.

**The author’s answer:**

We greatly appreciate your professional review of our study in such valuable time. At the beginning of the study, we found through reading literature that many scholars believe that the digital transformation of enterprises is a positive performance in response to the continuous maturity of “Artificial Intelligence, Blockchain, Cloud Computing and Big Data” technology development, and it is an excellent quasi-natural experiment for enterprises to gradually promote their own digital transformation behavior in batches. DID model is selected to further overcome the endogenous problem: through Treatment Group and Control Group, the difference before and after the implementation of digital transformation strategy, effectively eliminate the internal differences between individuals and the bias caused by the time trend unrelated to the experimental group, and the “net effect” of enterprise digital transformation on stock liquidity can be obtained. Therefore, we also used this DID model to test the endogeneity in the benchmark regression. The DID model you proposed should be based on external shocks, and it is not appropriate to build a quasi-natural experiment with the digital transformation behavior of enterprises as the impact point, which also reminds us that this approach is questionable. Therefore, we deleted the did-d model in the study with a rigorous scientific research attitude.

**3.** Model (5) is introduced to tackle the issue of omitted variables by examining the potential variation in the positive impact of the firm’s introduction of digital transformation on stock liquidity across different levels of digital transformation. Regardless of the appropriateness of the DID model setting, there is an error in Model (5), and the accurate model should be as follows:

$${Liquidity}_{i,t}=\gamma_{0}+\gamma_{1}{du}_{i}+\gamma_{2}{dt}_{t}+\gamma_{3}{DIG}_{i,t}+\gamma_{4}{du}_{i}*{dt}_{t}+\gamma_{5}{du}_{i}*{DIG}_{i,t}+\gamma_{6}{dt}_{t}*{DIG}_{i,t}+\gamma_{7}{dt}_{t}*{{dt}_{t}*DIG}_{i,t}+\sum Firm+\sum Year+\varepsilon_{i,t}$$

And ${du}_{i}$ and ${dt}_{t}$ is omitted due to the firm-fixed effects and year fixed effects, so the final model is:

$${Liquidity}_{i,t}=\gamma_{0}+\gamma_{1}{DIG}_{i,t}+\gamma_{2}{du}_{i}*{dt}_{t}+\gamma_{3}{du}_{i}*{DIG}_{i,t}+\gamma_{4}{dt}_{t}*{DIG}_{i,t}+\gamma_{5}{dt}_{t}*{{dt}_{t}*DIG}_{i,t}+\sum Firm+\sum Year+\varepsilon_{i,t}$$

where $\gamma_{5}$ is the coefficient of interest.

**The author's answer:**

We greatly appreciate your professional review of our study in such valuable time, pointing out our problems in building the DID model and helping us to learn the DID model of preparation, which is very important for our future research and learning. Thank you again.

**4.** In the mechanism analysis, financing constraint (FC), internal control (IC), and information disclosure (ID) are selected as the three channels. However, s given the relationship between stock liquidity and a firm’s information transparency, it is reasonable to analyze channels related to information disclosure. Conversely, the relevance of financing constraint (FC) and internal control (IC) to stock liquidity may require further clarification. For example, the statement in line 156 suggests that “by expanding financing channels and optimizing capital structure and share capital structure, enterprises can enhance information transparency and market recognition and ease financing constraints, which are conducive to improving stock liquidity.”. Is there any reference supporting this perspective? I recommend that the authors emphasize how financing constraints and internal control contribute to either the enhancement or reduction of stock liquidity from the perspective of information transparency. Otherwise, it is better to expand the analysis for the channels of information disclosure.

**The author’s answer:**

(1)We greatly appreciate your professional review of our study in such valuable time.We see you mentioned in the opinion of 156 lines of relevant description reference problem, in the process of research we refer to some literature expounds the paragraph, but after the manuscript found the article overall reference references and deleted some references to cause some paragraphs in the manuscript seems no literature support. In Revised manuscript “2.1 Digital transformation and stock liquidity”. We supplemented relevant references and use this as experience to avoid these problems in future studies.

(2)We greatly appreciate your professional review of our study in such valuable time.In Revised manuscript “2.2.1Digital transformation, financing constraints, and stock liquidity" and “2.2.2 Digital transformation, internal control, and stock liquidity”.In accordance with your suggestion, we supplement the discussion on financing constraints and internal control to improve or reduce stock liquidity from the perspective of information transparency, and further review the relationship between financing constraints and internal control and equity liquidity, so as to make up for the incomplete previous discussion and help readers better understand this study.

**5.** In Table 7, the direct effect and indirect effect of digital transformation on stock liquidity are 0.0078 (0.00641*1.224) and 0.0359, respectively. The sum of the direct effect and indirect effect is 0.0437 (0.0078+0.0359), which is considerably higher than the total effect of 0.0367 as presented in the baseline results. Please double check the results. In addition, the percentage of indirect effect 0.0008 (0.0367-0.0359) in total effect is 2.18%, which is relatively small.

**The author’s answer:**

We greatly appreciate your professional review of our study in such valuable time.The sum of the direct and indirect effects of the internal control (IC) mentioned in your review opinion is higher than the total effect in the benchmark regression, and we tested the agreement between the results and the ones described in the manuscript by stata software. At the same time, we also note that the indirect effect of internal control (IC) you mentioned is relatively small. In future research, we will continue to look for the greater indirect effect between digital transformation and stock liquidity in enterprises.

**6.** In the heterogeneity analysis, the moderators influencing the relationship between digital transformation and stock liquidity should be determinants of digital transformation. Kindly provide an explanation regarding how the financial technology level and degree of financial market development impact the firm’s digital transformation. Additionally, please include a detailed description of the measurement for these three moderators.

**The author’s answer:**

(1)We greatly appreciate your professional review of our study in such valuable time.In Revised manuscript “6.1Financial technology level”, we supplemented the impact of fintech on the digital transformation of enterprises from three aspects: financial resources, financial stability and innovation vitality. Meanwhile, we supplemented the calculation methods and references of the variable indicators of fintech.

(2)We greatly appreciate your professional review of our study in such valuable time.In Revised manuscript “6.2Degree of financial market development”, we supplement the impact of financial marketization degree on the digital transformation of enterprises from three perspectives of resource consumption, market competition and risk taking. At the same time, we supplemented the calculation methods of scalar indicators and references of financial marketization.

(3)We greatly appreciate your professional review of our study in such valuable time.In Revised manuscript “6.3Digital policy guidance",we supplement the way in which the digital policy-guided variable indicators are calculated.

**7.** In the heterogeneity analysis, the coefficients of $\mathrm{DIG}$ on $\mathrm{Liquidity}$ are both significant, as shown in Table 8. It is recommended to provide the statistics of the coefficients difference of $\mathrm{DIG}$ for these two groups.

**The author’s answer:**

We greatly appreciate your professional review of our study in such valuable time.According to your opinion, we chose Chow-test to test the difference between the DIG estimation coefficient of the two groups. The test results show that the difference in the DIG estimation coefficient of the two groups in fintech level, financial marketization degree and heterogeneity guided by digital policy is 0.027,0.016 and 0.068, respectively. The results show that the difference in dig coefficient of the three types of heterogeneity test results is significant.

8. In the further analysis, this paper examines the economic consequences of enterprise digital transformation in improving stock liquidity., that is, $\mathrm{Liquidity}$ is considered as the channel through which $\mathrm{DIG}$ impacts stock price crash risk and analyst forecast quality. While this analysis is sound, the indirect effect of $\mathrm{DIG}$ on stock price crash risk through $\mathrm{Liquidity}$ is -0.00020 (-0.0367*0.00536), and accounts for 32% (-0.00019/-0.00625) of the total effect, which is relatively small.

**The author’s answer:**

We greatly appreciate your professional review of our study in such valuable time.We performed regression testing again using stata software to test the results consistent with those described in the manuscript. We also focus on the small indirect effect of DIG through Liquidity, and we will explore the greater economic consequences of indirect effect in future research, which also provides the direction and requirements for our future research. I want to thank you again.

**9.** This paper needs a proofread.

**The author’s answer:**

We greatly appreciate your professional review of our study in such valuable time.After we received the review comments, we revised the language, format and font problems existing in the manuscript.

**10.** Others

**10.1** In Table 1, why stock return is defined as earnings per share?

**The author’s answer:**

We greatly appreciate your professional review of our study in such valuable time.We found that earnings per share can reflect the investment value of the company’s stock, can reflect the earnings per share for investors, can represent the company’s market performance, may affect the enterprise digital transformation and stock liquidity, thus choose earnings per share as a control variable, and in the variable description and rigorous determine the name of earnings per share. To avoid ambiguity we change Table 1 “stock return” to “earnings per share”, and change the variable definition to more detailed “The ratio of after-tax profit to the total number of shares”.

**10.2** Explain why the sample period spans from 2012 to 2021.

**The author’s answer:**

We greatly appreciate your professional review of our study in such valuable time.The concept of “digital transformation” was first proposed by IBM in 2012, in the study to ensure the continuity and adequacy of sample size during the study, according to the practice and the empirical test at home and abroad, and considering the huge impact of the novel coronavirus on Chinese enterprises since 2021, the node of the study period as 2021, the study period as 10 years, namely 2012-2021.To more clearly explain the sample study cycle, we added the relevant content in the revised manuscript “3.1Data and sample”.

**10.3** The paper has more than just the following errors in formatting; please carefully address them:

(1) Apostrophes seem in different font format from others.

**The author’s answer:**

We greatly appreciate your professional review of our study in such valuable time.We took the Apostrophes fonts format in the full text and unified all the different fonts.We found that there are two types of “ ’ ” and “ ' ” in the “Times New Roman” font. Most of the articles published by Plos One are “ ’ ”, so we unified the Apostrophe in the text as “ ’ ”.

(2) There should a space between “quality” and “(Manita et al., 2020)” in line 42, “Stock liquidity” and “(Liquidity)” in line 255, “Table” and “3” in line 320, et al. Please carefully double check.

**The author’s answer:**

We greatly appreciate your professional review of our study in such valuable time.We examined these issues and other parts of the manuscript and revised them. Also what you received is reviewing the manuscript may be our original submitted version. After the first two rounds of external review, ’“quality” and “(Manita et al., 2020)” in line 42’ has modified the text and corrected the problem. Meanwhile, similar issues arise elsewhere that we have revised in the latest revised manuscript.

(3) In line 175, 320, 516, and 523, “stock” seems in different font format from others.

**The author’s answer:**

We greatly appreciate your professional review of our study in such valuable time.We checked the full text of the manuscript and corrected all of the font formatting problems.

**10.4** All variables used in the paper should be describe in detail.

**The author's answer:**

We greatly appreciate your professional review of our study in such valuable time.Following your opinion, we supplement the detailed calculation method of all control variables in Table 1, and the calculation method of all variables is described in detail in the “Description of indicators and data” file in the Supporting Information “S1 Appendix”.

**Reviewer #5:**

**The author’s answer:**

We greatly appreciate your professional review of our study in such valuable time.

**Modification description of references**

In order to express the theoretical framework more clearly and concisely and enhance the theoretical foundation of this article, we have made modifications to the references in the revised manuscript.

**1. The following references have been added:**

Niu Y, Wang S, Wen W, Li S. Does digital transformation speed up dynamic capital structure adjustment? Evidence from China. Pacific-Basin Finance Journal. 2023;79:102016.

Tian J, Shao B. Financing constraints and information asymmetry of SMEs—The development of digital finance and financial risks of enterprises. Journal of the Knowledge Economy. 2023:1-21.

Brynjolfsson E, McElheran K. The rapid adoption of data-driven decision-making. American Economic Review. 2016;106(5):133-9.

Chen W, Cai W, Hu Y, Zhang Y, Yu Q. Gimmick or revolution: can corporate digital transformation improve accounting information quality? International Journal of Emerging Markets. 2022.

Brogaard J, Li D, Xia Y. Stock liquidity and default risk. Journal of Financial Economics. 2017;124(3):486-502.

Fang VW, Tian X, Tice S. Does stock liquidity enhance or impede firm innovation? The Journal of finance. 2014;69(5):2085-125.

Tsai C-H, Kuan-Jung P. The FinTech revolution and financial regulation: The case of online supply-chain financing. Asian Journal of Law and Society. 2017;4(1):109-32.

Alt R, Beck R, Smits MT. FinTech and the transformation of the financial industry. Springer; 2018. p. 235-43.

Demertzis M, Merler S, Wolff GB. Capital Markets Union and the fintech opportunity. Journal of financial regulation. 2018;4(1):157-65.

Li C, Yan X, Song M, Yang W. Fintech and corporate innovation: Evidence from Chinese NEEQ–listed companies. China Industrial Economics. 2020;1(8198):99999.

Love I. Financial development and financing constraints: International evidence from the structural investment model. The review of financial studies. 2003;16(3):765-91.

Zhao X, Wang Z, Deng M. Interest rate marketization, financing constraints and R&D investments: Evidence from China. Sustainability. 2019;11(8):2311.

Fan G, Wang X, Ma G. Contribution of marketization to China’s economic growth. Economic Research Journal. 2011;9(283):1997-2011.

**2. The following references were deleted:**

Zhao T, Yan N, Ji L. Digital transformation, life cycle and internal control effectiveness: Evidence from China. Finance Research Letters. 2023;58:104223. https://doi.org/10.1016/j.frl.2023.104223

Wen Z, Ye B. Analyses of mediating effects: the development of methods and models. Advances in psychological Science. 2014;22(5):731. https://doi.org/10.3724/SP.J.1042.2014.00731

Shipman JE, Swanquist QT, Whited RL. Propensity score matching in accounting research. The Accounting Review. 2017;92(1):213-44. https://doi.org/10.2308/accr-51449

Hua Z, Yu Y. Digital transformation and the impact of local tournament incentives: Evidence from publicly listed companies in China. Finance Research Letters. 2023;57:104204.

Peng Y, Tao C. Can digital transformation promote enterprise performance?—From the perspective of public policy and innovation. Journal of Innovation & Knowledge. 2022;7(3):100198. https://doi.org/10.1016/j.jik.2022.100198

Jiang K, Du X, Chen Z. Firms' digitalization and stock price crash risk. International Review of Financial Analysis. 2022;82:102196. https://doi.org/10.1016/j.irfa.2022.102196

Chiang T C, Zheng D. Liquidity and stock returns: Evidence from international markets[J]. Global Finance Journal, 2015, 27: 73-97. https://doi.org/10.1016/j.gfj.2015.04.005

Yonghong L, Jie S, Ge Z, Ru Z. The impact of enterprise digital transformation on financial performance—Evidence from Mainland China manufacturing firms. Managerial and Decision Economics. 2023;44(4):2110-24. https://doi.org/10.1002/mde.3805

Ding R, Hou W. Retail investor attention and stock liquidity. Journal of international financial markets, institutions and money. 2015;37:12-26. https://doi.org/10.1016/j.intfin.2015.04.001

Roll R. A simple implicit measure of the effective bid‐ask spread in an efficient market. The Journal of finance. 1984;39(4):1127-39. https://doi.org/10.1111/j.1540-6261.1984.tb03897.x

Jung H, Song C-K. Managerial perspectives on climate change and stock price crash risk. Finance Research Letters. 2023;51:103410. https://doi.org/10.1016/j.frl.2022.103410

Chen W, Zhang L, Jiang P, Meng F, Sun Q. Can digital transformation improve the information environment of the capital market? Evidence from the analysts' prediction behaviour. Accounting & Finance. 2022;62(2):2543-78. https://doi.org/10.1111/acfi.12873

Fang M, Nie H, Shen X. Can enterprise digitization improve ESG performance? Economic Modelling. 2023;118:106101. https://doi.org/10.1016/j.econmod.2022.106101

Le H, Gregoriou A. How do you capture liquidity? A review of the literature on low‐frequency stock liquidity. Journal of Economic Surveys. 2020;34(5):1170-86.

Fang M, Nie H, Shen X. Can enterprise digitization improve ESG performance? Economic Modelling. 2023;118:106101. https://doi.org/10.1016/j.econmod.2022.106101

Hope OK. Disclosure practices, enforcement of accounting standards, and analysts' forecast accuracy: An international study. Journal of accounting research. 2003;41(2):235-72. https://doi.org/10.1111/1475-679X.00102

**Data Provision Statement**

The data used in this study is provided in the supporting information in the form of an attachment. The support information file name is called: S1 Appendix.
