# Supplementary material for: Enterprise digital transformation’s impact on stock liquidity: A corporate governance perspective
Source: PLoS One. 2024 Mar 20;19(3):e0293818. doi: 10.1371/journal.pone.0293818 (PMC10954117; doi:10.1371/journal.pone.0293818)
Supplement: Attachment — Submitted filename: Referee Report for PONE-D-23-30283.docx [file pone.0293818.s004.docx]

**Referee Report for PONE-D-23-30283 “Enterprise Digital Transformation’s Impact on Stock Liquidity: A Corporate Governance Perspective”**

**Summary**

This study examines the impact, mechanism, and economic consequences of enterprise digital transformation on stock liquidity, yielding four key findings. First, it reveals that enterprise digital transformation effectively enhances stock liquidity. Second, it identifies financing constraints, internal control quality, and information disclosure as the three channels through which enterprise digital transformation improves stock liquidity. Third, it establishes that the relationship between digital transformation and stock liquidity is more pronounced for policy-guided firms, and those in high-fintech areas and developed financial markets. Finally, it demonstrates that enterprise digital transformation can reduce the risk of stock price crashes and improve the quality of analysts' forecasts by improving stock liquidity.

While the topic is interesting and shows substantive improvements after the revision, this paper still faces several methodological issues. Detailed comments are provided below.

**Main concerns**

**1.** The bid-ask spread is a commonly used proxy for stock liquidity, as employed by Fang et al. (2014) and Brogaard et al. (2017). It is advisable to enhance the robustness of the baseline results using bid-ask spread to measure stock liquidity.

**2.** The DID model should be based on exogenous shocks, which are not determined by the research objectives, namely the firms in this paper. Constructing a quasi-natural experiment using enterprise digital transformation behavior may not be appropriate, as digital transformation is a company's own development strategy. For instance, policies related to digital transformation could provide an ideal setting for the DID model.

**3.** Model (5) is introduced to tackle the issue of omitted variables by examining the potential variation in the positive impact of the firm's introduction of digital transformation on stock liquidity across different levels of digital transformation. Regardless of the appropriateness of the DID model setting, there is an error in Model (5), and the accurate model should be as follows:

$${Liquidity}_{i,t}=\gamma_{0}+\gamma_{1}{du}_{i}+\gamma_{2}{dt}_{t}+\gamma_{3}{DIG}_{i,t}+\gamma_{4}{du}_{i}*{dt}_{t}+\gamma_{5}{du}_{i}*{DIG}_{i,t}+\gamma_{6}{dt}_{t}*{DIG}_{i,t}+\gamma_{7}{dt}_{t}*{{dt}_{t}*DIG}_{i,t}+\sum Firm+\sum Year+\varepsilon_{i,t}$$

And ${du}_{i}$ and ${dt}_{t}$ is omitted due to the firm-fixed effects and year fixed effects, so the final model is:

$${Liquidity}_{i,t}=\gamma_{0}+\gamma_{1}{DIG}_{i,t}+\gamma_{2}{du}_{i}*{dt}_{t}+\gamma_{3}{du}_{i}*{DIG}_{i,t}+\gamma_{4}{dt}_{t}*{DIG}_{i,t}+\gamma_{5}{dt}_{t}*{{dt}_{t}*DIG}_{i,t}+\sum Firm+\sum Year+\varepsilon_{i,t}$$

where $\gamma_{5}$ is the coefficient of interest.

**4.** In the mechanism analysis, financing constraint (FC), internal control (IC), and information disclosure (ID) are selected as the three channels. However, s given the relationship between stock liquidity and a firm's information transparency, it is reasonable to analyze channels related to information disclosure. Conversely, the relevance of financing constraint (FC) and internal control (IC) to stock liquidity may require further clarification. For example, the statement in line 156 suggests that “*by expanding financing channels and optimizing capital structure and share capital structure, enterprises can enhance information transparency and market recognition and ease financing constraints, which are conducive to improving stock liquidity.*”. Is there any reference supporting this perspective? I recommend that the authors emphasize how financing constraints and internal control contribute to either the enhancement or reduction of stock liquidity from the perspective of information transparency. Otherwise, it is better to expand the analysis for the channels of information disclosure.

**5.** In Table 7, the direct effect and indirect effect of digital transformation on stock liquidity are 0.0078 (0.00641*1.224) and 0.0359, respectively. The sum of the direct effect and indirect effect is 0.0437 (0.0078+0.0359), which is considerably higher than the total effect of 0.0367 as presented in the baseline results. Please double check the results. In addition, the percentage of indirect effect 0.0008 (0.0367-0.0359) in total effect is 2.18%, which is relatively small.

**6.** In the heterogeneity analysis, the moderators influencing the relationship between digital transformation and stock liquidity should be determinants of digital transformation. Kindly provide an explanation regarding how the financial technology level and degree of financial market development impact the firm’s digital transformation. Additionally, please include a detailed description of the measurement for these three moderators.

**7.** In the heterogeneity analysis, the coefficients of $DIG$ on $Liquidity$ are both significant, as shown in Table 8. It is recommended to provide the statistics of the coefficients difference of $DIG$ for these two groups.

**8.** In the further analysis, this paper examines the economic consequences of enterprise digital transformation in improving stock liquidity., that is, $Liquidity$ is considered as the channel through which $DIG$ impacts stock price crash risk and analyst forecast quality. While this analysis is sound, the indirect effect of $DIG$ on stock price crash risk through $Liquidity$ is -0.00020 (-0.0367*0.00536), and accounts for 32% (-0.00019/-0.00625) of the total effect, which is relatively small.

**9.** This paper needs a proofread.

**Others**

**1.** In Table 1, why stock return is defined as earnings per share?

**2.** Explain why the sample period spans from 2012 to 2021.

**3.** The paper has more than just the following errors in formatting; please carefully address them:

(1) Apostrophes seem in different font format from others.

(2) There should a space between “quality” and “(Manita et al., 2020)” in line 42, “Stock liquidity” and “(Liquidity)” in line 255, “Table” and “3” in line 320, et al. Please carefully double check.

(3) In line 175, 320, 516, and 523, “stock” seems in different font format from others.

**4.** All variables used in the paper should be describe in detail.

**Reference**

[1] Brogaard, J., Li, D., & Xia, Y. (2017). Stock liquidity and default risk. Journal of Financial Economics, 124(3), 486-502.

[2] Fang, V. W., Tian, X., & Tice, S. (2014). Does stock liquidity enhance or impede firm innovation? The Journal of finance, 69(5), 2085-2125.
